# Supplementary material for: Curated and harmonised transcriptomics datasets of interstitial lung diseases
Source: Data Brief. 2025 Oct 14;63:112139. doi: 10.1016/j.dib.2025.112139 (PMC12581653; doi:10.1016/j.dib.2025.112139)
Supplement: Supplementary file 1 [file mmc1.zip › Supplementary_material/RNA-seq/fastQC reports/GSE138283/TRIMMED_SRR10212993_fastqc.html]

TRIMMED\_SRR10212993.fastq.gz FastQC Report 

FastQC Report

Thu 5 Oct 2023  
TRIMMED\_SRR10212993.fastq.gz

## Summary

- Basic Statistics
- Per base sequence quality
- Per sequence quality scores
- Per base sequence content
- Per sequence GC content
- Per base N content
- Sequence Length Distribution
- Sequence Duplication Levels
- Overrepresented sequences
- Adapter Content

## Basic Statistics

| Measure | Value |
| --- | --- |
| Filename | TRIMMED\_SRR10212993.fastq.gz |
| File type | Conventional base calls |
| Encoding | Sanger / Illumina 1.9 |
| Total Sequences | 80393559 |
| Sequences flagged as poor quality | 0 |
| Sequence length | 60-76 |
| %GC | 47 |

## Per base sequence quality

## Per sequence quality scores

## Per base sequence content

## Per sequence GC content

## Per base N content

## Sequence Length Distribution

## Sequence Duplication Levels

## Overrepresented sequences

| Sequence | Count | Percentage | Possible Source |
| --- | --- | --- | --- |
| CCACAAATTATGCAGTCGAGTTTCCCACATTTGGGGAAATCGCAGGGGTCAGCACATCCGGAGTGCAATGGATA | 550729 | 0.6850411983875474 | No Hit |
| CCCCACTACCACAAATTATGCAGTCGAGTTTCCCACATTTGGGGAAATCGCAGGGGTCAGCACATCCGGAGTGCA | 550264 | 0.6844627938414818 | No Hit |
| CCCACTACCACAAATTATGCAGTCGAGTTTCCCACATTTGGGGAAATCGC | 430404 | 0.5353712478383996 | No Hit |
| CCTTAGGCAACCTGGTGGTCCCCCGCTCCCGGGAGGTCACCATATTGATG | 374848 | 0.46626620921210865 | No Hit |
| CACAAATTATGCAGTCGAGTTTCCCACATTTGGGGAAATCGCAGGGGTCA | 349503 | 0.4347400517496681 | No Hit |
| CTCAGACCGCGTTCTCTCCCTCTCACTCCCCAATACGGAGAGAAGAACGATCATCAATGGCTGACGGCAGTTGC | 308562 | 0.38381433020025896 | No Hit |
| CTCCGTTTCCGACCTGGGCCGGTTCACCCCTCCTTAGGCAACCTGGTGGT | 272586 | 0.3390644765459382 | No Hit |
| CCCCTCCTTAGGCAACCTGGTGGTCCCCCGCTCCCGGGAGGTCACCATAT | 260752 | 0.3243443918187526 | No Hit |
| CCCTCCTTAGGCAACCTGGTGGTCCCCCGCTCCCGGGAGGTCACCATATT | 191260 | 0.23790463114091018 | No Hit |
| CCAGGCTGGAGTGCAGTGGCTATTCACAGGCGCGATCCCACTACTGATCA | 190491 | 0.2369480868485994 | No Hit |
| GTCGAGTTTCCCACATTTGGGGAAATCGCAGGGGTCAGCACATCCGGAGTGCAATGGATAAGCCTCGCCCTGGGA | 173825 | 0.2162175703653075 | No Hit |
| CTGGAGTCTTGGAAGCTTGACTACCCTACGTTCTCCTACAAATGGACCTTGAGAGCTTGTTTGGAGGTTCTAGC | 161399 | 0.2007611082375393 | No Hit |
| CCTCCTTAGGCAACCTGGTGGTCCCCCGCTCCCGGGAGGTCACCATATTGATGCCGAACTTAGTGCGGACACCCG | 148128 | 0.184253566880899 | No Hit |
| CTCAGGCTGGAGTGCAGTGGCTATTCACAGGCGCGATCCCACTACTGATC | 139353 | 0.1733385133503046 | No Hit |
| CCACTACCACAAATTATGCAGTCGAGTTTCCCACATTTGGGGAAATCGCA | 137528 | 0.1710684309920898 | No Hit |
| CACTACCACAAATTATGCAGTCGAGTTTCCCACATTTGGGGAAATCGCAG | 137014 | 0.17042907628955697 | No Hit |
| CTTAGGCAACCTGGTGGTCCCCCGCTCCCGGGAGGTCACCATATTGATGC | 135365 | 0.1683779169423262 | No Hit |
| GTCCCCCACTACCACAAATTATGCAGTCGAGTTTCCCACATTTGGGGAAA | 124069 | 0.1543270400555348 | No Hit |
| CTCCTTAGGCAACCTGGTGGTCCCCCGCTCCCGGGAGGTCACCATATTGA | 123274 | 0.15333815486387412 | No Hit |
| GTGGCTATTCACAGGCGCGATCCCACTACTGATCAGCACGGGAGTTTTGA | 122997 | 0.15299359989772315 | No Hit |
| CAGGCTGGAGTGCAGTGGCTATTCACAGGCGCGATCCCACTACTGATCAG | 120525 | 0.1499187266979933 | No Hit |
| GTCTGGAGTCTTGGAAGCTTGACTACCCTACGTTCTCCTACAAATGGACC | 104265 | 0.1296932257968577 | No Hit |
| GCTCCGTTTCCGACCTGGGCCGGTTCACCCCTCCTTAGGCAACCTGGTGGTCCCCCGCTCCCGGGAGGTCACCAT | 101568 | 0.12633847942967671 | No Hit |
| CTACCACAAATTATGCAGTCGAGTTTCCCACATTTGGGGAAATCGCAGGG | 93743 | 0.11660511260609821 | No Hit |

## Adapter Content

Produced by FastQC (version 0.11.7)
